# Association between albumin-corrected calcium and all-cause mortality in patients with heart failure: a retrospective study

**DOI:** 10.3389/fcvm.2025.1552807

**Published:** 2025-03-06

**Authors:** Xiongda Yao, Yurong Leng, Junda Cao

**Affiliations:** ^1^ Department of Cardiology, Jiangxi Medical College, The Second Affiliated Hospital, Nanchang University, Nanchang, Jiangxi, China; ^2^ Department of Stomatology, Jiangxi Medical College, The Affiliated Stomatological Hospital, Nanchang University, Nanchang, Jiangxi, China; ^3^ Cardiovascular Medicine, Jiujiang City Key Laboratory of Cell Therapy, Jiujiang No. 1 People’s Hospital, Jiujiang, Jiangxi, China

**Keywords:** albumin-corrected calcium, heart failure, MIMIC-IV database, all-cause mortality, calcium

## Abstract

**Background:**

Heart failure (HF), a global health challenge, is a leading cause of mortality in hospitalized patients. Early and accurate prognostic evaluation in these patients is vital for guiding clinical management. Our aim was to explore the association between albumin-corrected calcium (ACC) and mortality in hospitalized patients.

**Methods:**

This retrospective cohort study utilized data from the Medical Information Mart for Intensive Care IV (MIMIC-IV) database. Patients were stratified into three groups based on ACC levels. The association between ACC and clinical outcomes in HF patients was analyzed using Cox proportional hazards regression and restricted cubic spline models.

**Results:**

A total of 4,737 heart failure patients were included. Multifactorial Cox regression revealed that elevated ACC levels were significantly associated with increased 30-day and 180-day mortality. Restricted cubic spline analysis demonstrated a U-shaped relationship between ACC levels and mortality, with an inflection point at 9.18. Patients with ACC levels above 9.18 exhibited an 20.4% higher risk of 30-day mortality [Hazard ratio (HR): 1.204, 95% (Confidence interval) CI: 1.009–1.437] and a 20.8% higher risk of 180-day mortality (HR: 1.208, 95% CI: 1.019–1.431) compared to those with ACC below 9.18.

**Conclusions:**

The observed U-shaped association between ACC levels and 30- and 180-day mortality in HF patients highlights the potential utility of ACC as a prognostic marker.

## Introduction

End-stage clinical symptoms of Heart failure (HF) have made it a major worldwide health concern. The World Disease Survey estimates that approximately 5 out of every 1,000 person-years in Europe currently suffer from HF (1). In China, the inpatient mortality rate for patients with HF is 4.1%, while the prevalence of HF is 0.9% (2). These figures underscore the urgent need for early and accurate prognostic assessment to improve clinical outcomes.

Calcium is necessary for various physiological processes, such as muscle contraction, neurotransmitter and hormone secretion, blood coagulation, and bone mineralization. Serum calcium is present in three forms: roughly 50% as ionized calcium, 40% bound to albumin, and 10% complexed with anions (3). In order to account for albumin's binding effect on calcium ions, ACC, which is frequently used to estimate ionized calcium levels, modifies the total calcium concentration based on serum albumin levels. Clinicians frequently use ACC as a proxy for free calcium levels (4). Due to frequent use of diuretics and water and sodium retention, patients with HF are more vulnerable to electrolyte abnormalities (5). Changes in free calcium levels can impair cardiomyocyte function and influence membrane potential, potentially deteriorating the prognosis for these patients. However, there is still little data examining the connection between ACC levels and HF prognosis, especially in individuals who are very sick.

Therefore, we aimed to evaluate the association between baseline ACC and all-cause mortality in patients admitted to the intensive care unit (ICU) with HF. The findings are anticipated to yield new insights into early risk stratification and strategies for prognostic improvement in this patient population.

## Method

### Study design

This retrospective cohort study utilized health-related data from the MIMIC-IV database (version 3.0), a robust resource curated by the Massachusetts Institute of Technology (MIT) Laboratory of Computational Physiology (6). The database contains extensive, high-quality medical records of patients hospitalized in the intensive care unit (ICU) at Beth Israel Deaconess Medical Center (BIDMC). The original data collection was approved by the BIDMC Institutional Review Board (Protocol #2001-P-001699/14), and patients were exempted from informed consent because the data were completely de-identified and the study was retrospective. MIMIC-IV ensures patient privacy through multiple layers of processing, following the Health Insurance Portability and Accountability Act (HIPAA) Safe Harbor Method of de-identification, which ensures that all 18 categories of potential identifiers are processed, with a very low risk of re-identification (<0.1% as assessed by a third party). Author Xiongda Yao completed HIPAA compliance training (Certificate #13971739), signed a Data Use Agreement promising not to attempt to re-identify patients or share raw data, and received authorization to extract and analyze data. The research adhered to the STROBE guidelines (Supplementary File S1) (7).

### Population

**Figure 1:**
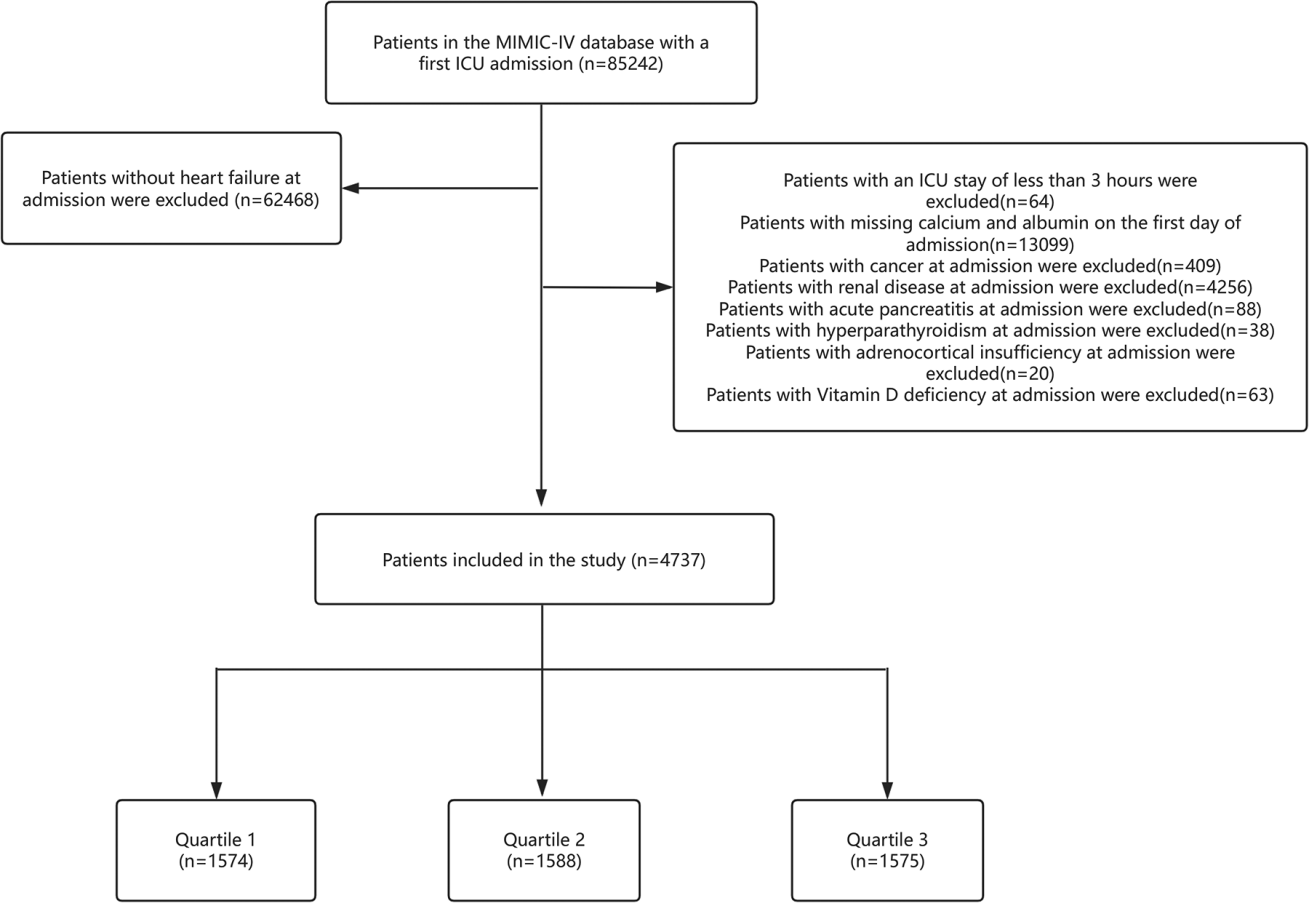
Flow of included patients through the trial.

Consistent with previous studies (8), the diagnosis of the patient's disease was determined by International Classification of Diseases ICD-9 or ICD-10 discharge diagnosis codes, as detailed in Supplementary Table S1. Inclusion criteria were patients 18 years of age or older whose discharge diagnosis included heart failure. Exclusion criteria were as follows (1) patients admitted to the intensive care unit for less than 3 h; (2) patients with insufficient data on serum calcium or serum albumin levels on the first day of admission; (3) patients with comorbid renal disease; (4) patients with comorbid malignancies; (5) patients with comorbid acute pancreatitis; (6) patients with comorbid hyperparathyroidism; (7) patients with comorbid adrenal insufficiency; (8) patients with comorbid vitamin D deficiency. A total of 4,737 patients met the inclusion criteria. Participants were divided into three groups according to ACC tertiles (Figure 1).

### Data collection

Data was retrieved from the PostgreSQL database management system through the use of Structured Query Language. Covariates were categorized into five primary groups: (1) Demographic characteristics: age, gender, race, weight, marital status; (2) Vital signs: heart rate, oxygen saturation, body temperature, respiratory rate, blood pressure; (3) Laboratory parameters: red blood cells (RBC), white blood cells (WBC), hemoglobin, platelets, mean red blood cell volume, red blood cells distribution width (RDW), glutamic oxaloacetic aminotransferase, glutamic alanine aminotransferase, bilirubin, serum albumin, blood urea nitrogen (BUN), serum creatinine, blood glucose, anion gap, serum calcium, serum sodium, serum potassium, serum chloride, N-terminal pro-brain natriuretic peptide (NT-pro BNP); (4) Medications administered: angiotensin-converting enzyme inhibitors, angiotensin II receptor antagonists, norepinephrine, dobutamine, dopamine, diuretic; (5) Comorbidities: respiratory failure, hypertension, diabetes mellitus, atrial fibrillation, chronic obstructive pulmonary disease (COPD), cardiac shock, stroke, acute myocardial infarction. Furthermore, disease severity scores upon admission were evaluated, including the Oxford Acute Severity of Illness (OASIS) Score, Sepsis-Related Organ Failure Assessment Score (SOFA), Acute Physiology and Chronic Health (APSIII) Score III, Simplified Acute Physiology (SAPS-II) Score II, Glasgow Coma Score (GCS), and Charleston Comorbidity Index (CCI) (9, 10). The period of follow-up lasted from the day of admission to the day of death. The ACC index is determined by the formula ACC = total serum calcium (mg/dl) + 0.8 × [4.0—serum albumin (g/dl)] (4). Scores for illness severity and all laboratory parameters were acquired within 24 h of ICU admission.

Variables with over 20% missing values were excluded from the analysis to mitigate potential bias. Due to the absence of NT-pro BNP data in many cases, we resorted to referring to other literature to supplement the missing information. Among the less than 20% missing values, no variables were missing except for the serologic indicators. Given that the proportion of missing values did not exceed 20%, Random Forest Multiple Interpolation was used for all serological indicator, with the “mice” package in R software (11).

### Clinical outcome

The primary outcome of the study was all-cause mortality at 30 and 180 days. The secondary outcomes were all-cause mortality at 14 and 90 days.

### Statistical analysis

Continuous variables were expressed as mean ± standard deviation (SD) or median (interquartile range), depending on their distribution, while categorical variables were presented as proportions. The normality of continuous data was evaluated using the Kolmogorov–Smirnov test. For normally distributed data, continuous variables were compared using the *t*-test or analysis of variance (ANOVA). For non-normally distributed data, the Mann–Whitney *U*-test or Kruskal-Walli's test was applied. Kaplan–Meier survival analysis was performed to evaluate the incidence of endpoint events across groups stratified by different ACC index levels, and differences were assessed using the log-rank test. Cox proportional hazards regression models were used to calculate HRs and 95% CIs for the association between ACC index levels and endpoint events, while adjusting for potential confounding variables. Confounding variables were identified using *P*-values <0.05 from univariate analyses, with clinically and prognostically significant variables included in multivariate models: Model 1 (uncorrected); Model 2 (adjusted for age, gender, and race); and Model 3 (further adjusted for RBC, WBC, platelet count, sodium, potassium, respiratory failure, hypertension, diabetes, atrial fibrillation, acute myocardial infarction, cardiogenic shock, stroke, GCS, and CCI). The study examined the nonlinear relationship between baseline ACC index levels and in-hospital all-cause mortality at 30 and 180 days using a restricted cubic spline regression model with three knots. ACC was incorporated into the model as either a continuous or ordinal variable, with the second quartile of the ACC index serving as the reference group. To evaluate the consistency of the ACC index's predictive value for the primary outcome, additional analyses were stratified by race, gender, age (≥60 or <60 years), respiratory failure, hypertension, diabetes mellitus, atrial fibrillation, acute myocardial infarction, cardiogenic shock, and stroke. The likelihood ratio test was used to evaluate interactions between the ACC index and stratification variables. To avoid overfitting the Cox regression model, we performed a sensitivity analysis using the least absolute shrinkage and selection operator (LASSO) regression and 10-fold cross-validation to select the most significant variables among the adjusted variables, and these selected variables were included in a multifactor Cox regression analysis to assess the robustness of our findings. A two-sided *P* value of <0.05 was considered statistically significant. All statistical analyses were performed using R software (version 4.4.2).

## Results

In this study, 4,737 individuals with severe congestive HF were involved. There were 2,471 (52.16%) male patients, with a median age of 72.65 years [interquartile range (IQR): 62.16–82.52 years]. All subjects had a median ACC index of 9.18 (IQR: 8.82–9.58). The in-hospital mortality rate was 16.38% (Table 1).

**Table 1:** Baseline characteristics of patients grouped according to ACC index quartiles.

Variable	Total (*n* = 4,737)	Q1 (6. 74–8.94) (*n* = 1,574)	Q2 (8.94–9.42) (*n* = 1,588)	Q3 (9.42–20.94) (*n* = 1,575)	*P* value
Demographics					
Age, years	72.65 (62.16,82.52)	71.49 (60.91,82.03)	72.70 (62.28,82.59)	73.71 (63.78,82.91)	<0.01
Gender, *n* (%)					<0.001
Female	2,266 (47.84)	693 (44.03)	727 (45.78)	846 (53.71)	
Male	2,471 (52.16)	881 (55.97)	861 (54.22)	729 (46.29)	
Race, *n* (%)					<0.001
White	3,401 (71.80)	1,161 (73.76)	1,125 (70.84)	1,115 (70.79)	
Asian	136 (2.87)	49 (3.11)	42 (2.64)	45 (2.86)	
Black	516 (10.89)	119 (7.56)	187 (11.78)	210 (13.33)	
Other	684 (14.44)	245 (15.57)	234 (14.74)	205 (13.02)	
Marital Status, *n* (%)					0.13
Married	1,873 (39.54)	618 (39.26)	664 (41.81)	591 (37.52)	
Divorced	402 (8.49)	130 (8.26)	128 (8.06)	144 (9.14)	
Single	1,167 (24.64)	391 (24.84)	382 (24.06)	394 (25.02)	
Widowed	806 (17.01)	256 (16.26)	254 (15.99)	296 (18.79)	
Other	489 (10.32)	179 (11.37)	160 (10.08)	150 (9.52)	
Weight Mean, kg	83.02 ± 27.37	83.02 ± 25.32	84.72 ± 27.99	81.31 ± 28.59	<0.01
Vital signs					
HR Mean, beats/min	87.50 ± 17.70	87.97 ± 17.64	86.50 ± 17.46	88.03 ± 17.95	0.02
SPO2 Mean, %	96.24 ± 3.06	96.28 ± 2.71	96.38 ± 2.31	96.06 ± 3.92	<0.01
Temperature Mean,°C	36.81 ± 0.55	36.86 ± 0.59	36.82 ± 0.50	36.75 ± 0.56	<0.001
RR Mean, times/min	20.63 ± 4.02	20.64 ± 4.11	20.54 ± 3.89	20.72 ± 4.06	0.43
SBP Mean, mmHg	114.17 ± 16.32	113.10 ± 15.77	114.95 ± 16.33	114.45 ± 16.81	<0.01
DBP Mean, mmHg	63.67 ± 11.35	63.35 ± 11.01	64.16 ± 11.33	63.49 ± 11.71	0.10
Laboratory indicators					
RBC Max, 10^−9^/L	3.66 ± 0.69	3.65 ± 0.69	3.69 ± 0.68	3.64 ± 0.70	0.11
WBC Max, 10^−9^/L	13.25 ± 9.52	13.14 ± 8.85	12.56 ± 7.74	14.04 ± 11.52	<0.001
Platelet Max, 10^−9^/L	213.34 ± 102.09	197.40 ± 96.83	217.49 ± 98.68	225.08 ± 108.44	<0.001
Hemoglobin Max, g/dl	10.67 ± 1.94	10.76 ± 1.97	10.70 ± 1.91	10.55 ± 1.94	0.01
MCV (fL)	91.05 ± 7.05	91.18 ± 6.80	90.72 ± 7.14	91.25 ± 7.20	0.07
RDW (%)	15.82 ± 2.38	15.60 ± 2.26	15.76 ± 2.36	16.11 ± 2.47	<0.001
ALT Max, U/L	28.00 (17.00,65.00)	31.00 (18.00,77.15)	28.00 (16.00,57.00)	28.00 (16.00,60.80)	<0.001
AST Max, U/L	42.00 (25.00,102.00)	48.00 (27.00,128.75)	39.00 (23.00,88.25)	42.00 (24.00,89.00)	<0.001
Bilirubin (umol/L)	1.29 ± 2.39	1.24 ± 1.84	1.27 ± 2.14	1.35 ± 3.02	0.39
Albumin Max, g/dl	3.33 ± 0.63	3.46 ± 0.60	3.37 ± 0.60	3.16 ± 0.66	<0.001
BUN Max, mg/dl	29.82 ± 18.95	28.31 ± 18.30	29.25 ± 18.61	31.90 ± 19.74	<0.001
Creatinine Max, mg/dl	1.31 ± 0.87	1.31 ± 0.94	1.27 ± 0.82	1.33 ± 0.85	0.20
Glucose Max, mg/dl	158.17 ± 78.09	155.00 ± 74.57	154.05 ± 75.75	165.49 ± 83.23	<0.001
Anion gap (mmol/L)	14.83 ± 4.32	14.85 ± 3.96	14.54 ± 4.06	15.10 ± 4.86	<0.01
Calcium Max, mg/dl	8.71 ± 0.85	8.12 ± 0.57	8.68 ± 0.49	9.34 ± 0.92	<0.001
Sodium Max, mmol/L	138.94 ± 5.29	138.37 ± 5.28	138.77 ± 5.01	139.68 ± 5.49	<0.001
Potassium Max, mmol/L	4.35 ± 0.69	4.28 ± 0.67	4.30 ± 0.63	4.45 ± 0.76	<0.001
Chloride Max, mEq/L	103.25 ± 6.58	103.77 ± 6.57	102.81 ± 6.22	103.16 ± 6.91	<0.001
ACC	9.18 (8.82,9.58)	8.64 (8.38,8.80)	9.18 (9.08,9.30)	9.78 (9.58,10.14)	<0.001
Medication situation					
ACEI/ARB					0.01
No	2,775 (58.58)	906 (57.56)	900 (56.68)	969 (61.52)	
Yes	1,962 (41.42)	668 (42.44)	688 (43.32)	606 (38.48)	
Epinephrine					<0.001
No	2,887 (60.95)	944 (59.97)	1,036 (65.24)	907 (57.59)	
Yes	1,850 (39.05)	630 (40.03)	552 (34.76)	668 (42.41)	
Dobutamine					0.23
No	4,397 (92.82)	1,473 (93.58)	1,475 (92.88)	1,449 (92.00)	
Yes	340 (7.18)	101 (6.42)	113 (7.12)	126 (8.00)	
Dopamine					0.28
No	4,473 (94.43)	1,486 (94.41)	1,510 (95.09)	1,477 (93.78)	
Yes	264 (5.57)	88 (5.59)	78 (4.91)	98 (6.22)	
Diuretic					0.25
No	880 (18.58)	308 (19.57)	275 (17.32)	297 (18.86)	
Yes	3,857 (81.42)	1,266 (80.43)	1,313 (82.68)	1,278 (81.14)	
Comorbidities					
Respiratory failure, *n* (%)					0.22
No	2,561 (54.06)	859 (54.57)	878 (55.29)	824 (52.32)	
Yes	2,176 (45.94)	715 (45.43)	710 (44.71)	751 (47.68)	
Hypertension, *n* (%)					<0.001
No	1,361 (28.73)	506 (32.15)	442 (27.83)	413 (26.22)	
Yes	3,376 (71.27)	1,068 (67.85)	1,146 (72.17)	1,162 (73.78)	
Diabetes, *n* (%)					<0.001
No	3,086 (65.15)	1,117 (70.97)	999 (62.91)	970 (61.59)	
Yes	1,651 (34.85)	457 (29.03)	589 (37.09)	605 (38.41)	
Atrial fibrillation, *n* (%)					0.13
No	2,427 (51.23)	832 (52.86)	783 (49.31)	812 (51.56)	
Yes	2,310 (48.77)	742 (47.14)	805 (50.69)	763 (48.44)	
COPD, *n* (%)					0.03
No	4,101 (86.57)	1,389 (88.25)	1,373 (86.46)	1,339 (85.02)	
Yes	636 (13.43)	185 (11.75)	215 (13.54)	236 (14.98)	
Cardiogenic shock, *n* (%)					0.21
No	4,012 (84.69)	1,338 (85.01)	1,360 (85.64)	1,314 (83.43)	
Yes	725 (15.31)	236 (14.99)	228 (14.36)	261 (16.57)	
Stroke, *n* (%)					0.02
No	4,212 (88.92)	1,425 (90.53)	1,389 (87.47)	1,398 (88.76)	
Yes	525 (11.08)	149 (9.47)	199 (12.53)	177 (11.24)	
AMI, *n* (%)					0.15
No	3,760 (79.38)	1,226 (77.89)	1,263 (79.53)	1,271 (80.70)	
Yes	977 (20.62)	348 (22.11)	325 (20.47)	304 (19.30)	
Disease Severity Score					
SOFA score	5.23 ± 3.60	5.35 ± 3.55	4.85 ± 3.53	5.50 ± 3.69	<0.001
APSIII score	49.17 ± 20.87	48.09 ± 20.26	46.93 ± 20.02	52.52 ± 21.89	<0.001
SAPSII score	39.32 ± 14.15	38.78 ± 13.59	37.77 ± 13.73	41.41 ± 14.85	<0.001
OASIS score	33.45 ± 8.74	33.39 ± 8.57	32.66 ± 8.64	34.30 ± 8.95	<0.001
GCS score	13.68 ± 2.61	13.78 ± 2.57	13.74 ± 2.55	13.52 ± 2.71	0.01
CCI score	5.70 ± 2.01	5.49 ± 2.03	5.76 ± 2.05	5.86 ± 1.94	<0.001
Length of stay (LOS)					
Los in hospital	8.71 (5.13,14.83)	8.53 (5.07,13.62)	8.73 (5.00,15.00)	8.87 (5.29,16.07)	0.01
Los in ICU	2.70 (1.38,5.24)	2.83 (1.51,5.30)	2.58 (1.32,5.06)	2.65 (1.33,5.34)	0.20
Outcomes					
14-day mortality, *n* (%)					<0.001
No	4,146 (87.52)	1,388 (88.18)	1,427 (89.86)	1,331 (84.51)	
Yes	591 (12.48)	186 (11.82)	161 (10.14)	244 (15.49)	
30-day mortality, *n* (%)					<0.001
No	4,020 (84.86)	1,353 (85.96)	1,385 (87.22)	1,282 (81.40)	
Yes	717 (15.14)	221 (14.04)	203 (12.78)	293 (18.60)	
90-day mortality, *n* (%)					<0.001
No	3,962 (83.64)	1,340 (85.13)	1,365 (85.96)	1,257 (79.81)	
Yes	775 (16.36)	234 (14.87)	223 (14.04)	318 (20.19)	
180-day mortality, *n* (%)					<0.001
No	3,961 (83.62)	1,339 (85.07)	1,365 (85.96)	1,257 (79.81)	
Yes	776 (16.38)	235 (14.93)	223 (14.04)	318 (20.19)	

ALT, alanine aminotransferase; AST, aspartate transaminase; HR, heart rate; RR, respiratory rate; SBP, systolic blood pressure; DBP, diastolic blood pressure; SPO2, pulse blood oxygen saturation; APSIII, acute physiology score; SAPSII, simplified acute physiology score; OASIS, oxford acute severity of illness score; SOFA, sequential organ failure assessment; ICU, intensive care unit.

### Baseline characteristics

Table 1 shows the baseline characteristics of patients with HF based on ACC index tertiles. Based on their ACC index levels at hospital admission, the enrolled patients were divided into three groups: quartile (Q)1 (6. 74–8.94), Q2 (8.94–9.42), and Q3 (9.42–20.94). In each quartile, the median ACC index was 8.64 (IQR: 8.38–8.80), 9.18 (IQR: 9.08–9.30), and 9.78 (IQR: 9.58–10.14). Compared to patients in the lower tertiles, those in the highest ACC index tertile (Q3) were generally older, had higher illness severity scores at admission, and exhibited higher prevalences of COPD, diabetes, hypertension, and stroke. Furthermore, patients in Q3 showed increased BUN, creatinine, RDW, WBC, platelet, sodium, potassium, and anionic gaps. Individuals in higher tertiles experienced longer hospital stays (8.53 days vs. 8.73 days vs. 8.87 days, *P* < 0.001) and increased hospital 180-day mortality rates (14.94% vs. 14.04% vs. 20.09%, *P* < 0.001) compared to those in the lower tertiles of the ACC index.

### Study outcomes

**Figure 2:**
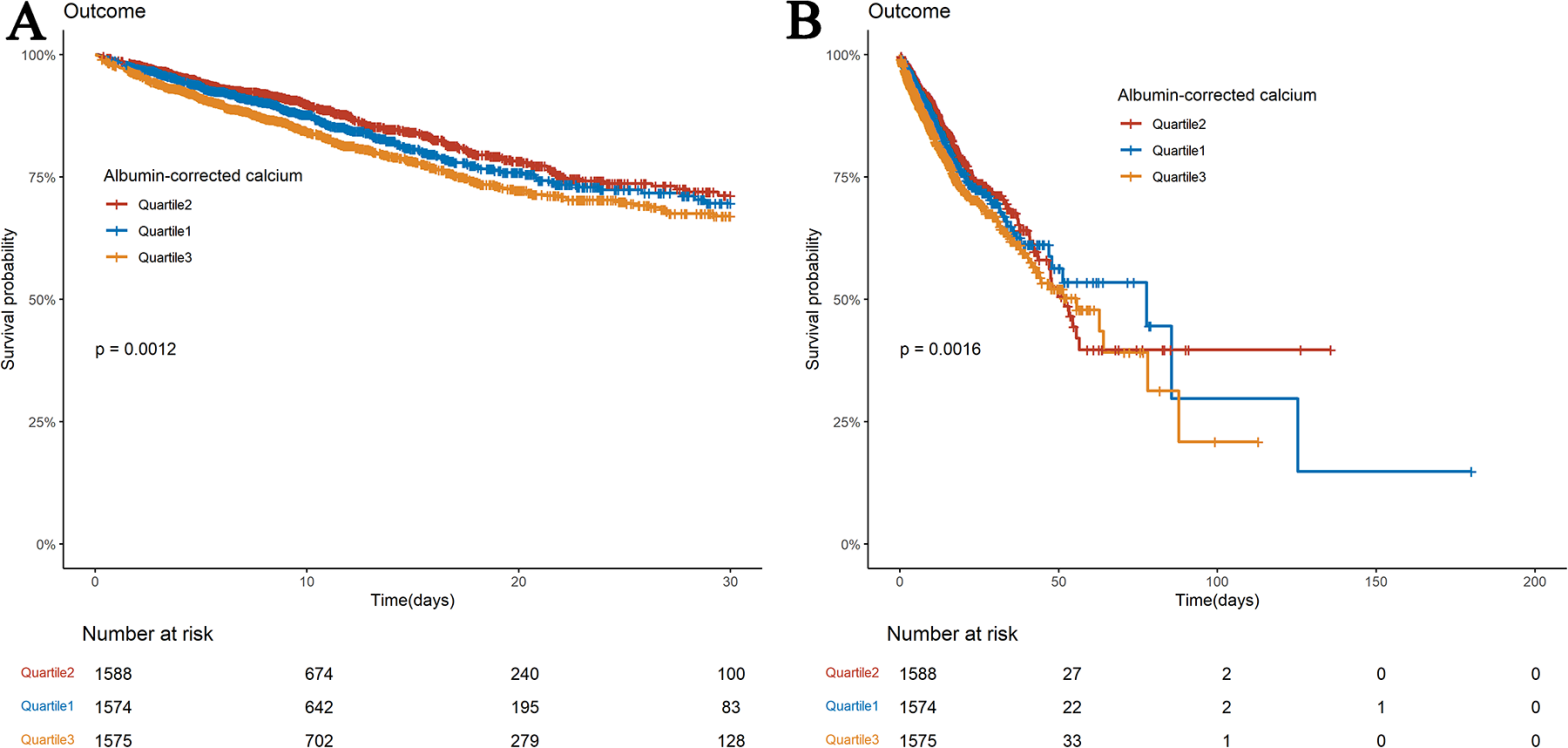
Kaplan–meier survival analysis curves for all-cause mortality. Kaplan–Meier curves of 30-day **(A)** and 180-day **(B)** all-cause mortality stratified by ACC index.

Kaplan–Meier survival curves illustrate differences in survival among the three ACC groups for 30- and 180-day mortality (Figure 2). Differences in mortality at 14 and 90 days are seen in Supplementary Figure S1. The survival rates at 14, 30, 90, and 180 days were substantially lower for patients in the highest ACC index group (Q3) than for those in the lowest ACC index group (Q1) (log-rank *P* < 0.05). However, at all-time points (14, 30, 90, and 180 days), no significant differences in survival were observed between the lowest ACC index group (Q1) and the intermediate group (Q2).

### Relationship between ACC and clinical outcomes of patients with HF

Two Cox regression models examined the ACC index's independent mortality effect (Tables 2, 3). After age, gender, and race adjustments (Model 2), the HRs and 95% CIs for ACC index categories (Q1, Q2, and Q3) for 30-day all-cause mortality were: HRs were 1.17 (0.973–1.407), 1 (reference), and 1.343 (1.129–1.597). Subsequent adjustments for age, gender, race, RBC, WBC, platelets, sodium, potassium, respiratory failure, hypertension, diabetes, atrial fibrillation, acute myocardial infarction, cardiogenic shock, stroke, GCS, and CCI (Model 3) resulted in the following hazard ratios for 30-day all-cause mortality: 1.149 (0.955–1.384), 1.00 (reference), and 1.204 (1.009–1.437), respectively. In Model 2, the hazard ratios for 180-day all-cause mortality were 1.147 (95% CI: 0.960–1.370), 1.00 (reference), and 1.319 (95% CI: 1.117–1.558), respectively. In Model 3, the HRs were 1.145 (0.957–1.370), 1.00 (reference), and 1.208 (1.019–1.431). The findings suggest that patients with an ACC index of 9.42 or higher had a significantly elevated risk of all-cause mortality at both 30 and 180 days compared to those in Q2. Similar trends for all-cause mortality at 14 and 90 days are presented in Supplementary Tables S1 and S2.

**Table 2:** Cox proportional hazard models for 30-day all-cause mortality.

Variables	Model 1		Model 2		Model 3	
	HR (95% CI)	*P*	HR (95% CI)	*P*	HR (95% CI)	*P*
ACC quantile						
1	1.152 (0.952,1.394)	0.146	1.202 (0.992, 1.456)	0.060	1.186 (0.977, 1.439)	0.085
2	1.00 (Reference)		1.00 (Reference)		1.00 (Reference)	
3	1.39 ( 1.162,1.663)	<0.001	1.397 (1.167, 1.673)	<0.001	1.248 (1.038, 1.500)	0.019

HR, hazard ratio, CI, confidence interval; ACC, albumin-corrected calcium.

Model 1: Crude.

Model 2: Adjust: age, sex, race.

Model 3: model 2 plus RBC, WBC, Platelet, Sodium, Potassium, Respiratory failure, Hypertension, Diabetes, Atrial fibrillation, Acute myocardial infarction, Cardiogenic shock, Stroke, Glasgow Coma Scale, Charlson Comorbidity Index.

**Table 3:** Cox proportional hazard models for 180-day all-cause mortality.

Variables	Model 1		Model 2		Model 3	
	HR (95% CI)	*P*	HR (95% CI)	*P*	HR (95% CI)	*P*
ACC quantile						
1	1.126 (0.938,1.353)	0.203	1.179 (0.980, 1.418)	0.081	1.183 (0.981, 1.425)	0.078
2	1.00 (Reference)		1.00 (Reference)		1.00 (Reference)	
3	1.357 (1.143,1.610)	<0.001	1.373 (1.155, 1.631)	<0.001	1.251 (1.048, 1.493)	0.013

HR, hazard ratio; CI, confidence Interval; ACC, albumin-corrected calcium.

Model 1: Crude.

Model 2: Adjust: age, sex, race.

Model 3: model 2 plus RBC, WBC, Platelet, Sodium, Potassium, Respiratory failure, Hypertension, Diabetes, Atrial fibrillation, Acute myocardial infarction, Cardiogenic shock, Stroke, Glasgow Coma Scale, Charlson Comorbidity Index.

### The detection of nonlinear relationships

**Figure 3:**
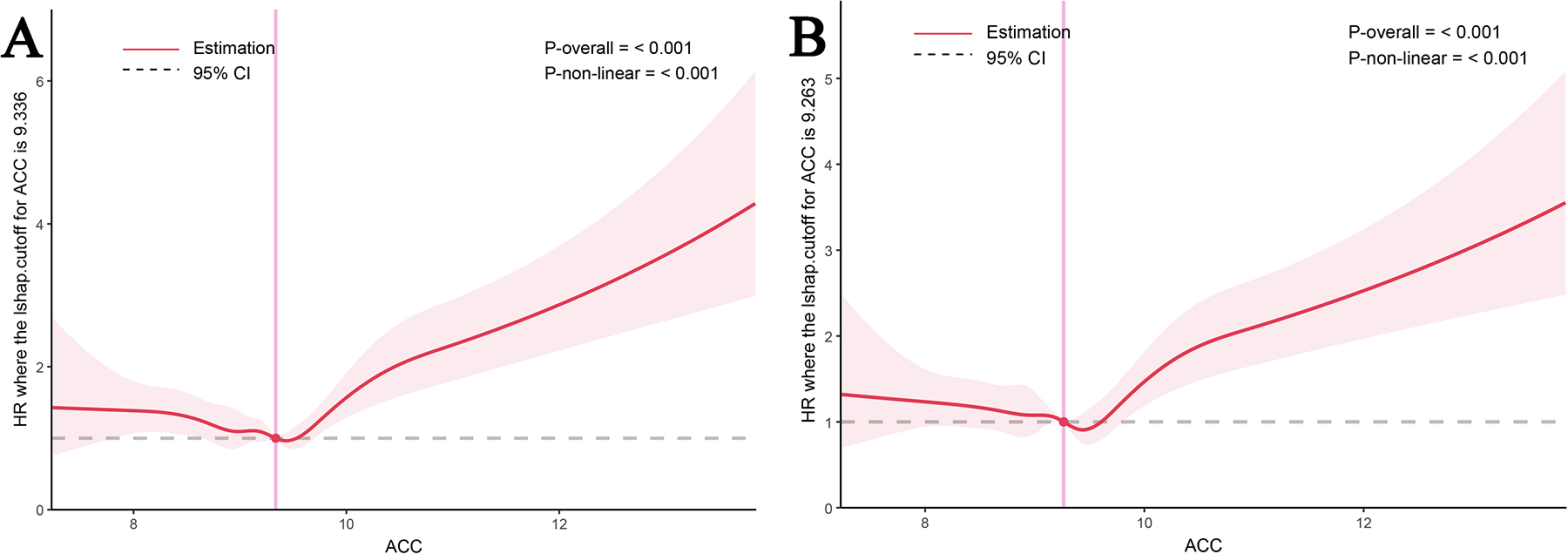
RCS of ACC index with all-cause mortality. RCS of ACC index with 30-day **(A)** and 180-day **(B)** all-cause mortality.

A nonlinear relationship between the ACC index and all-cause mortality at multiple time points (14, 30, 90, and 180 days) was identified using restricted cubic spline (RCS) curve analysis. At 30 and 180 days, the ACC index specifically showed a U-shaped relationship with mortality (Figures 3A,B), and same patterns were seen for mortality at 14 and 90 days (Supplementary Figures S2A,B). Tables 4, 5, along with Supplementary Tables 3 and S4, illustrate that both the Cox proportional hazards model and the two-stage Cox proportional hazards model, employed to assess this nonlinear relationship, yielded statistically significant results (log-likelihood ratio test *P* < 0.05). The analysis revealed an inflection point at an ACC index of 9.18 for both 30day and 180day all-cause mortality rates. The risk of 30 day all-cause death rose by 37.4% for every unit rise in ACC when the ACC index surpassed 9.18 (*P* < 0.001; 95% CI: 1.278–1.478). There was a 33.4% increase in risk for 180 day all-cause death for every unit rise in the ACC index over 9.18 (*P* < 0.001; 95% CI: 1.242–1.432).

**Table 4:** Threshold effect analysis of ACC on 30-day all-cause mortality in HF patients.

30-day mortality	HR (95% CI), *P*-value
Fitting by the standard linear regression	1.259 (1.173, 1.352) < 0.0001
Fitting by the two-piecewise linear regression	
Inflection point	9.18
ACC < 9.18	0.75 (0.572, 0.982) 0.037
ACC ≥ 9.18	1.374 (1.278, 1.478) < 0.0001
*P* for Log-likelihood ratio	<0.0001

**Table 5:** Threshold effect analysis of ACC on 180-day all-cause mortality in HF patients.

180-day mortality	HR (95% CI), *P*-value
Fitting by the standard linear regression	1.234 (1.154, 1.319) < 0.0001
Fitting by the two-piecewise linear regression	
Inflection point	9.18
ACC < 9.18	0.812 (0.622, 1.061) 0.127
ACC ≥ 9.18	1.334 (1.242, 1.432) < 0.0001
*P* for Log-likelihood ratio	<0.001

ACC, Albumin-corrected calcium; The inflection of threshold effect analysis of ACC on 180-day all-cause mortality was 9.18.

### Stratified analyses

**Figure 4:**
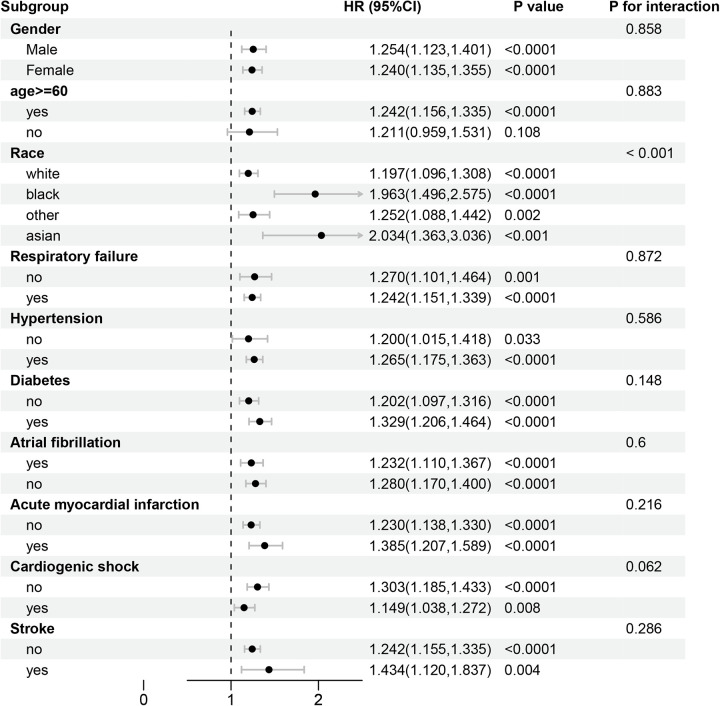
Forest plots of stratified analyses of ACC index and 30-day all-cause mortality.

**Figure 5:**
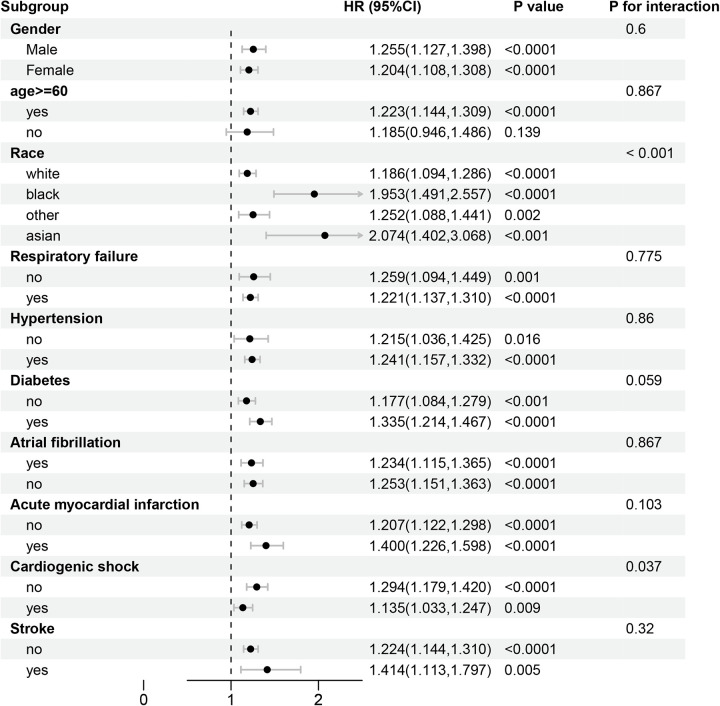
Forest plots of stratified analyses of ACC index and 180-day all-cause mortality.

A subgroup analysis based on age, gender, race, respiratory failure, hypertension, diabetes mellitus, atrial fibrillation, acute myocardial infarction, cardiac shock, and stroke was performed to examine whether the association between ACC and 14, 30, 90, and 180 day all-cause mortality remained consistent across conditions. In the subgroup aged ≥60 years, the hazard ratios (HRs) for all-cause mortality at 14, 30, 90, and 180 days were statistically significant (*P* < 0.05) (Figures 4, 5) (Supplementary Figures S3, S4). In the subgroup of individuals younger than 60 years, the hazard ratios were not statistically significant (*P* > 0.05). The association between ACC levels and all-cause mortality at 30 and 180 days was statistically significant (*P* < 0.05), independent of patients' histories of respiratory failure, hypertension, diabetes mellitus, atrial fibrillation, myocardial infarction, cardiac shock, or stroke.

Interactive analyses indicated no significant differences (*P* > 0.05) in 30 and 180 day all-cause mortality related to gender, age (≤60 years or >60 years), or the presence of respiratory failure, hypertension, diabetes mellitus, atrial fibrillation, acute myocardial infarction, or stroke. Significant differences in 30 and 180 day all-cause mortality were noted among patients of varying races (*P* < 0.05). No significant difference was observed in the 30 day all-cause mortality between patients with cardiogenic shock and those without. In contrast, a statistically significant difference emerged in the 180 day all-cause mortality (*P* < 0.05).

### Sensitivity analyses

In the LASSO regression model, non-zero factors were selected as predictors of all-cause mortality in this study (Supplementary Figures S5A,B), and then, we further incorporated these potential factors associated with all-cause mortality into the multivariate Cox regression model. Age, race, leukocytes, platelets, potassium, respiratory failure, hypertension, cardiac shock, GCS, and CCI were finally screened to be significantly associated with all-cause mortality (Supplementary Table S4). The model results remained stable after incorporating these variables into the Cox regression model (Supplementary Tables S5, S6).

## Discussion

These are the first retrospective studies to examine ACC and all-cause mortality in HF patients. A U-shaped correlation exists between ACC levels and mortality at 14, 30, 90, and 180 days. The findings suggest that an increase in ACC levels beyond a specific threshold is correlated with a heightened risk of mortality. This study's insights may inform treatment strategies aimed at reducing mortality risk in patients with severe HF.

A significant amount of death in HF patients admitted to hospitals is ascribed to electrolyte imbalances (12–14), which include variations in calcium, sodium, and potassium levels. The Get With The Guidelines-Heart Failure (GWTG-HF) system for prognostic assessment in HF patients does not include aberrant blood calcium values, however these disturbances are very common in this population (15). Alterations in calcium metabolism can affect neuromuscular excitability, resulting in cardiac arrhythmias and dysfunction across multiple organs (16). Previous studies have shown that disturbances in calcium homeostasis are common in critically ill patients and that both elevated and low levels are associated with increased adverse outcomes. A 2018 study by Shiyovich et al. identified a U-shaped between serum calcium levels (<9.12 mg/dl and >9.86 mg/dl) and in-hospital mortality in patients with acute myocardial infarction, suggesting that serum calcium serves as an independent predictor of mortality (17). A study that included 44,886 critically ill patients demonstrated a nonlinear U-shaped relationship between serum calcium concentration and in-hospital mortality, with patients who deviated from the reference range (8.6–9.0 mg/dl) having a significantly higher risk of death (18). Similarly, an analysis of 91 patients with sepsis showed that serum calcium levels were significantly associated with increased 30-day mortality (19). A cohort study that included 119 patients with sepsis showed that 28-day mortality was significantly higher in patients with hypocalcemia compared to those with normal calcium (20). These findings suggest that serum calcium is an independent predictor of mortality. However, total serum calcium is affected by blood pH and serum albumin levels, potentially diminishing its reliability as a clinical marker. Ionized calcium offers a more precise indication of calcium status; however, its measurement is complex and influenced by various factors. To present, no studies have particularly looked into the role of ACC in HF patients. This study retrospectively analyzed data from 4,737 patients with HF in the MIMIC database, revealing a nonlinear U-shaped association between ACC levels and the risk of all-cause mortality at 30 and 180 days. The curve exhibited an initial decline followed by an increase, with an inflection point at approximately 9.18, indicating the lowest mortality risk. Deviations from the 9.18 threshold, whether above or below, were associated with an increased risk of in-hospital mortality.

Our findings remained consistent across most subgroups. Notably, within the ethnic subgroups, we observed significantly higher 30-day and 180-day in-hospital mortality rates in Asian and African populations compared to European populations. We hypothesize that these disparities may be attributable to differences in socioeconomic status, accessibility to healthcare resources, and genetic backgrounds (21). The association between ACC and all-cause mortality in patients with heart failure may vary across populations and healthcare systems. Racial disparities in calcium metabolism have garnered significant scholarly attention. A prevailing body of research has indicated that serum calcium levels are generally lower in African populations, a phenomenon that can be attributed, at least in part, to genetic polymorphisms in vitamin D-binding proteins (e.g., GC gene variants) (22). The dietary profile of Asian populations, characterized by low calcium intake and a high prevalence of vitamin D deficiency, is postulated to result in lower baseline calcium levels (23). This, in turn, may influence the strength of the association between ACC and mortality. The allocation of resources and the implementation of treatment strategies within disparate healthcare systems have the potential to exert a substantial influence on the correlation between ACC and mortality. In high-income countries, the earlier diagnosis and intervention of ACC may serve to diminish its prognostic significance, as hyper- or hypocalcemia can be addressed in a timely manner. Conversely, in low-income countries, where healthcare resources are scarce, ACC abnormalities may be identified at a later stage or remain uncorrected, thereby amplifying its association with mortality (24). The present study also demonstrated that the 180-day in-hospital mortality rate was higher in the non-cardiogenic shock subgroup than in the cardiogenic shock subgroup. At first glance, this may appear inconsistent with the well-documented high morbidity and mortality associated with cardiogenic shock. However, this result can be better understood by examining the differences in patient group characteristics. The cardiogenic shock subgroup typically comprises acutely ill patients with a clearer etiology and more defined interventions, such as mechanical circulatory support, vasoactive medications, early percutaneous coronary intervention, or intra-aortic balloon counter pulsation, which often lead to more aggressive treatment and monitoring (25, 26). In contrast, the non-cardiogenic shock subgroup often includes patients with multiple comorbidities, significant metabolic disturbances, and atypical symptoms. These patients may experience progressive deterioration due to chronic cardiac insufficiency, malnutrition, or electrolyte imbalances. Furthermore, delays in treatment and low recognition rates are common in this subgroup, contributing to their higher mortality rates.

Several mechanisms may explain the relationship between calcium homeostasis and HF. Calcium ions have a critical role in maintaining normal cardiac function, including regulation of myocardial contraction and diastole, cardiomyocyte signaling, and maintenance of intracellular metabolic homeostasis (27, 28). Disturbances in calcium homeostasis can adversely affect the heart's ability to contract, ultimately leading to impaired cardiac function (29). HF may worsen due to arrhythmias caused by abnormal calcium signaling (30). A calcium imbalance can have detrimental effects on vascular and cardiac tissues. For instance, calcium deposition on coronary artery walls causes calcification, a risk factor for HF (31, 32). Furthermore, calcium ions have been demonstrated to play a pivotal role in the coagulation cascade. Dysregulation of blood calcium has been shown to result in impaired platelet function and effects on the coagulation cascade, which may contribute to bleeding events and a poor prognosis in patients with heart failure (33–35). Abnormalities in calcium homeostasis have been demonstrated to be significantly associated with an increased incidence of acute kidney injury, hypotension, disseminated intravascular coagulation, and organ failure. These adverse events may lead to an increased risk of death in patients with HF (36–38).

## Comparison with previous studies and further research

A retrospective cohort study of 7,063 patients with HF and diabetes mellitus revealed a U-shaped association between serum calcium levels and in-hospital mortality (39). Similarly, Jensen et al. analyzed a cohort of 2,729 Danish patients and hypothesized a U-shaped association between serum calcium levels and 30-day mortality in patients with chronic HF (40). These conclusions are consistent with our findings. In contrast to previous studies, our study used ACC to more accurately measure the biologically active calcium ion concentration and included a longer follow-up period. This approach provides a clearer understanding of the relationship between calcium homeostasis and long-term prognosis in patients with HF. These findings offer valuable insights for clinicians, aiding in risk assessment and treatment optimization.

## Clinical implications

The findings indicate the necessity of incorporating ACC into the management strategy for patients with HF. Clinicians must be cognizant of patients' ACC status during the diagnostic process and treatment planning, and they must provide early intervention and management of HF patients with abnormal ACC to enhance prognosis.

## Limitations

This study has several limitations to consider. First, our study was unable to fully account for medications influencing calcium metabolism (e.g., vitamin D supplements, bisphosphonates) due to incomplete pharmacologic data in the dataset. This residual confounding represents an important limitation, and findings should be interpreted with this caveat in mind. Second, as a retrospective cohort study, despite adjustments for confounders, the analysis may still be affected by residual confounding factors. Third, we used ICD-9 and ICD-10 codes to identify patients and therefore highly relied on the accuracy of ICD codes, in addition some key parameters such as NYHA cardiac function class and BNP could not be obtained from ICD codes. Finally, our study population consisted primarily of participants from the United States, which may limit the generalization of the findings to other populations.

## Conclusion

Our findings reveal that ACC was an independent risk factor for all-cause death at 30 and 180 days in heart failure patients. In the future, more research is required to validate this.

## Data Availability

The original contributions presented in the study are included in the article/Supplementary Material, further inquiries can be directed to the corresponding author.
