# Supplementary material for: Association between albumin-corrected calcium and all-cause mortality in patients with heart failure: a retrospective study
Source: Front Cardiovasc Med. 2025 Mar 6;12:1552807. doi: 10.3389/fcvm.2025.1552807 (PMC11922875; doi:10.3389/fcvm.2025.1552807)
Supplement: Supplementary file 1 [file Supplementaryfile1.docx]

**Supplemental Table 1.** Disease diagnosis of this study and corresponding ICD codes.

| **Disease diagnosis** | **ICD-9 codes** | **ICD-10 codes** |
| --- | --- | --- |
| Heart failure | 428.0-428.4, 428.9 | I50.0- I50.4, I50.8, I50.9 |
| Acute myocardial infarction | 410.0-410.5 | I21.0-I21.4, I21.9, I22, I23 |
| Chronic pulmonary disease | 490, 505, 4168, 4169, 5064, 5081, 5088 | J40, J47, J60, J67, I278, I279, J684, J701, J703 |
| Hypertension | 401.0, 401.1, 401.9, 405.01, 405.09, 405.11, 405.19, 405.91, 405.9 | I10, I15, I15.0-I15.2, I15.8, I15.9 |
| Diabetes | 250.0-250.9 | E10-E14.9 |
| Renal disease | 580-586 | N00, N03, N10, N11, N17, N18, N19 |
| Malignant cancer | 140, 172, 174.0, 195.8, 200, 208, 238.6 | C43, C88, C00-C26, C30-C34, C37-C41, C45-C58, C60-C76, C81-C85, C90-C97 |
| Respiratory failure | 518.81- 518.83 | J96.0-J96.2, J80 |
| Cardiogenic shock | 785.51 | R57.0 |
| Stroke | 430-437 | I60- I69, G45 |
| Acute pancreatitis | 577.0-577.2 | K85.0- K85.3, K85.8, K85.9 |
| Hyperparathyroidism | 252.0, 252.1, 252.2, 252.8, 252.9 | E21.0, E21.1, E21.2, E21.8, E21.9 |
| Vitamin D deficiency | 268.0, 268.2, 268.9 | E55.0, E55.9 |
| Adrenal insufficiency | 255.4 | E27.1-E27.4, E23.0, E24 |

**Supplemental Table 2.** Cox proportional hazard models for 14-day all-cause mortality

| **Variables** | Model1 | | Model2 | | Model3 | |
| --- | --- | --- | --- | --- | --- | --- |
|  | HR (95%CI) | *P* | HR (95%CI) | *P* | HR (95%CI) | *P* |
| ACC quantile |  |  |  |  |  |  |
| 1 | 1.19(0.963,1.469) | 0.107 | 1.226(0.992, 1.516) | 0.060 | 1.193(0.963, 1.478) | 0.106 |
| 2 | 1.00 (Reference) |  | 1.00 (Reference) |  | 1.00 (Reference) |  |
| 3 | 1.498(1.228,1.828) | <0.0001 | 1.487(1.218, 1.816) | <0.0001 | 1.279(1.043, 1.569) | 0.018 |

HR: Hazard Ratio, CI: Confidence Interval, ACC: Albumin-corrected calcium

Model1: Crude

Model2: Adjust: age, sex, race

Model3: model2 plus RBC, WBC, Platelet, Sodium, Potassium, Respiratory failure, Hypertension, Diabetes, Atrial fibrillation, Acute myocardial infarction, Cardiogenic shock, Stroke, Glasgow Coma Scale, Charlson Comorbidity Index

**Supplemental Table 3.** Cox proportional hazard models for 90-day all-cause mortality

| **Variables** | Model1 | | Model2 | | Model3 | |
| --- | --- | --- | --- | --- | --- | --- |
|  | HR (95%CI) | *P* | HR (95%CI) | *P* | HR (95%CI) | *P* |
| ACC quantile |  |  |  |  |  |  |
| 1 | 1.122(0.934,1.348) | 0.220 | 1.175(0.976, 1.413) | 0.088 | 1.177(0.976, 1.419) | 0.088 |
| 2 | 1.00 (Reference) |  | 1.00 (Reference) |  | 1.00 (Reference) |  |
| 3 | 1.354(1.141,1.607) | 0.002 | 1.369(1.152, 1.627) | <0.001 | 1.247(1.045, 1.488) | 0.014 |

HR: Hazard Ratio, CI: Confidence Interval, ACC: Albumin-corrected calcium

Model1: Crude

Model2: Adjust: age, sex, race

Model3: model2 plus RBC, WBC, Platelet, Sodium, Potassium, Respiratory failure, Hypertension, Diabetes, Atrial fibrillation, Acute myocardial infarction, Cardiogenic shock, Stroke, Glasgow Coma Scale, Charlson Comorbidity Index

**Supplemental Table 4.** The prediction model with multivariate cox regression

| **Variable** | **Multivariate analysis**  **HR (95%CI)** | **P** |
| --- | --- | --- |
| Age | 1.029(1.021-1.037) | <0.001 |
| Race |  |  |
| White | Reference |  |
| Black | 1.136(0.847-1.541) | 0.413 |
| Asian | 1.018(0.641-1.641) | 0.942 |
| Other | 1.611(1.291-2.009) | <0.001 |
| RBC | 0.882(0.777-1.002) | 0.053 |
| WBC | 1.013(1.008-1.018) | <0.001 |
| Platelet | 0.999(0.998-1.000) | 0.042 |
| Potassium | 1.199(1.073-1.339) | 0.001 |
| Respiratory failure | 1.444(1.200-1.739) | <0.001 |
| Hypertension | 0.717(0.597-0.861) | <0.001 |
| Cardiogenic shock | 2.048(1.683-2.493) | <0.001 |
| GCS | 0.946(0.923-0.971) | <0.001 |
| CCI | 1.078(1.024-1.135) | 0.004 |
| ACC | 1.131(1.045-1.224) | 0.002 |

**Supplemental Table 5.** Sensitivity analysis for 30- and 180-day all-cause mortality

| **Variables** | 30-day mortality | | 180-day mortality | |
| --- | --- | --- | --- | --- |
|  | HR (95%CI) | *P* | HR (95%CI) | *P* |
| ACC quantile |  |  |  |  |
| 1 | 1.229(0.986,1.533) | 0.067 | 1.221(1.015, 1.470) | 0.034 |
| 2 | 1.00 (Reference) |  | 1.00 (Reference) |  |
| 3 | 1.259(1.022,1.551) | <0.001 | 1.329(1.118, 1.581) | <0.001 |

HR: Hazard Ratio, CI: Confidence Interval, ACC: Albumin-corrected calcium

Model adjust for: age, race, WBC, Platelet, Potassium, Respiratory failure, Hypertension, Cardiogenic shock, Glasgow Coma Scale, Charlson Comorbidity Index

**Supplemental Table 6.** Sensitivity analysis for 14- and 90-day all-cause mortality

| **Variables** | 14-day mortality | | 90-day mortality | |
| --- | --- | --- | --- | --- |
|  | HR (95%CI) | *P* | HR (95%CI) | *P* |
| ACC quantile |  |  |  |  |
| 1 | 1.265(0.989,1.617) | 0.061 | 1.204(0.974,1.488) | 0.086 |
| 2 | 1.00 (Reference) |  | 1.00 (Reference) |  |
| 3 | 1.321(1.045,1.669) | 0.020 | 1.258(1.031,1.534) | 0.024 |

HR: Hazard Ratio, CI: Confidence Interval, ACC: Albumin-corrected calcium

Model adjust for: age, race, WBC, Platelet, Potassium, Respiratory failure, Hypertension, Cardiogenic shock, Glasgow Coma Scale, Charlson Comorbidity Index
